# CAPER as a therapeutic target for triple negative breast cancer

**DOI:** 10.18632/oncotarget.25719

**Published:** 2018-07-13

**Authors:** Mallory C. Campbell, Laura Pontiggia, Ashley Y. Russell, Roland Schwarting, Jeanette Camacho, Jean-Francois Jasmin, Isabelle Mercier

**Affiliations:** ^1^ Department of Pharmaceutical Sciences, Philadelphia College of Pharmacy, University of the Sciences, Philadelphia, PA 19104, USA; ^2^ Department of Mathematics, Physics and Statistics, Misher College of Arts and Sciences, University of the Sciences, Philadelphia, PA 19104, USA; ^3^ Department of Pathology, Cooper University Hospital, Camden, NJ 08103, USA; ^4^ Program in Personalized Medicine and Targeted Therapeutics, University of the Sciences, Philadelphia, PA 19104, USA

**Keywords:** breast cancer, oncology targets, triple negative breast cancer, DNA repair

## Abstract

Breast cancers (BCas) that lack expression of the estrogen receptor (ER), progesterone receptor (PR), and human epidermal growth factor receptor 2 (HER2) are referred to as triple negative breast cancers (TNBCs) and have the poorest clinical outcome. Once these aggressive tumors progress to distant organs, the median survival decreases to 12 months. With endocrine therapies being ineffective in this BCa subtype, highly toxic chemo- and radiation therapies are the only options. A better understanding of the functional role(s) of molecular targets contributing to TNBC progression could help in the design and development of new treatments that are more targeted with less toxicity. CAPER (Co-activator of AP-1 and ER) is a nuclear transcriptional co-activator that was recently involved in ER-positive BCa progression, however its role in hormone-independent cancers remains unknown. Our current report demonstrates that CAPER expression is upregulated in human TNBC specimens compared to normal breast tissue and that its selective downregulation through a lentiviral-mediated shRNA knockdown approach resulted in decreased cell numbers in MDA-MB-231 and BT549 TNBC cell lines without affecting the growth of non-tumorigenic cell line MCF-10A. Concordant with these observations, CAPER knockdown was also associated with a decrease in DNA repair proteins leading to a marked increase in apoptosis, through caspase-3/7 activation without any changes in cell cycle. Collectively, we propose CAPER as an important signaling molecule in the development of TNBC linked to DNA repair mechanisms, which could lead to new therapeutic modalities for the treatment of this aggressive cancer.

## INTRODUCTION

Breast cancer (BCa) has become one of the most common cancer among women, with nearly 200,000 new cases diagnosed each year [1]. This diverse disease ultimately becomes a major cause of death in women of all ages and ethnicities. The most curable subtypes of BCa express at least one of the receptor targets linked to oncogenesis; estrogen receptor (ER), progesterone receptor (PR), and human epidermal growth factor receptor 2 (HER2). Triple negative breast cancer (TNBC) is defined by its lack of ER, PR, and HER2 receptors expression. These TNBCs are poorly differentiated and transition quickly to a more aggressive metastatic course than any other BCa subtypes, leading to worst prognosis and shortest survival rates [2, 3]. The lack of targetable receptors within TNBC results in highly cytotoxic systemic chemotherapy and radiation treatments, placing these patients at a clinical disadvantage [4–6]. Targeted therapy for TNBC patients has become an important research focus and many clinical trials are underway to specifically target DNA repair pathways [7].

TNBCs have been shown to exhibit upregulation of DNA repair genes involved in repair pathways [8], however, there are still many unknown pathways involved in the molecular and cellular functions of DNA repair that could change our understanding and treatment of TNBC. CAPER, (Co-activator of AP-1 and ER), also known as RNA binding motif 39 (RBM39), is a nuclear transcriptional co-activator of the activator protein 1 (AP-1) and ER that can also facilitate pre-mRNA processing [9]. A recent study revealed that CAPER is directly involved in alternative splicing of DNA repair genes as assessed in MCF-7 cells in which CAPER was silenced using siRNA technology [10]. CAPER protein expression was demonstrated to be upregulated in human invasive ductal carcinoma (IDC) specimens when compared to matching normal breast tissue [11] and lentiviral-mediated knockdown of CAPER expression in ER-positive MCF-7 cells markedly reduced tumor cell growth both *in vitro* and *in vivo* [12]. However, the role of CAPER in hormone-independent TNBC development and its involvement in DNA repair pathways remains completely unknown.

Our current report shows that CAPER expression is significantly higher in TNBC human specimens when compared to normal breast tissue and that its targeted decrease in TNBC cells results in lower cell numbers *in vitro*. Mechanistically, loss of CAPER protein impairs the functional repair of DNA through a decrease in RAD51, c-Abl, and Rb protein expressions, leading to the induction of apoptosis via caspase-3/7-mediated pathways. Novel therapeutic targets within TNBC that could fine-tune DNA repair pathways are interesting avenues for the treatment of this cancer subtype.

## RESULTS

### Upregulation of CAPER protein expression in human breast cancer specimens

**Figure 1:**
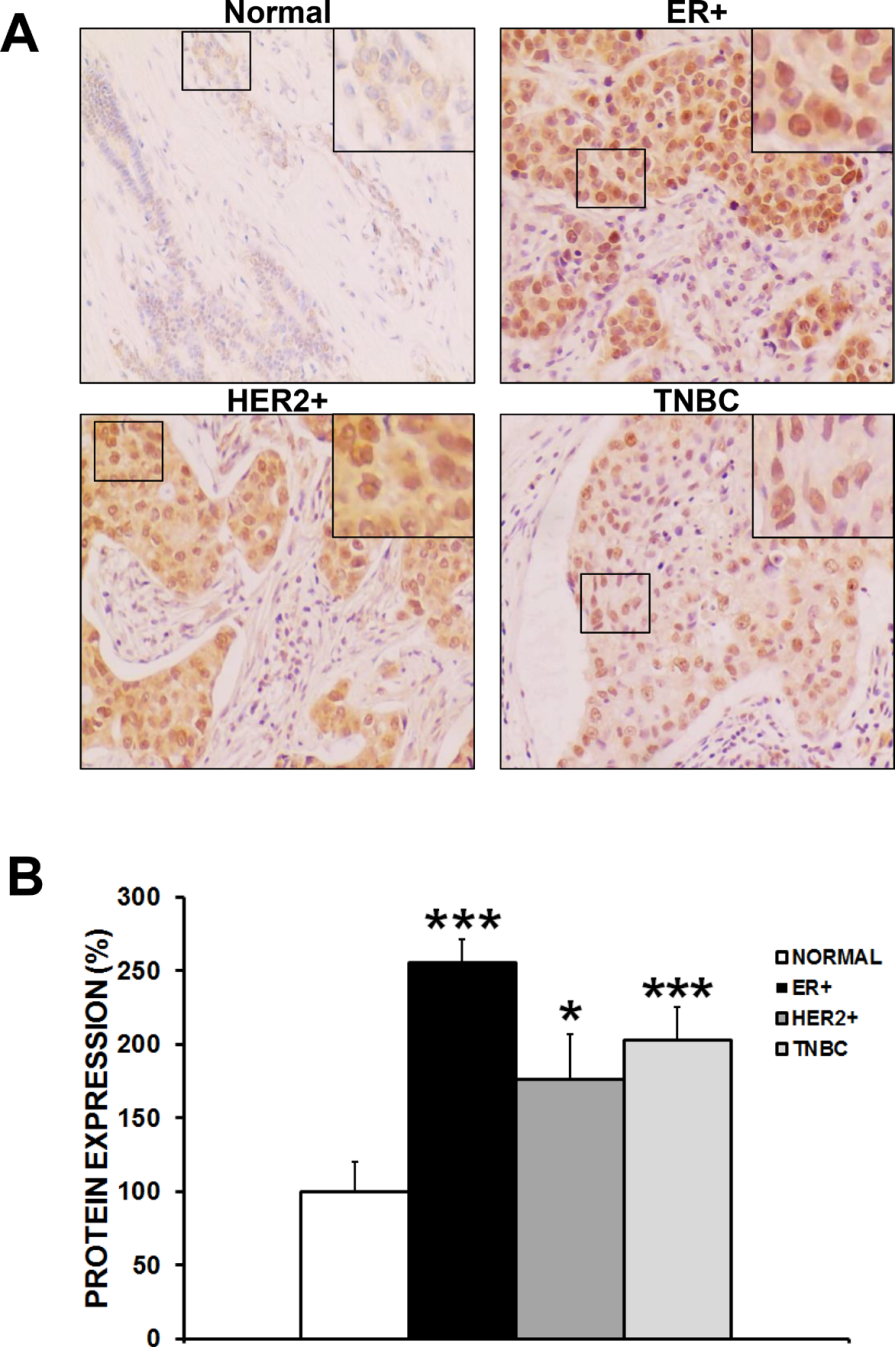
CAPER expression is induced in human breast cancer specimens (**A**) Immunohistochemical analysis reveals upregulated nuclear CAPER expression in ER+, HER2+ and TNBC breast cancer specimens when compared to normal benign breast specimens. Brown staining corresponds to cells positive for 3,3′-Diaminobenzidine (DAB) staining and intensity is proportional to CAPER expression. Pictures were taken at 20× an EVOS microscope. (**B**) Semiquantitative analyses as assessed by histoscore (H-score) method show a significant increase in nuclear CAPER protein levels in ER+ (2.5-fold, *p <* 0.001, *n* = 192), HER2+ (1.7-fold, *p <* 0.05, *n* = 48) and TN breast cancer patients (2-fold, *p <* 0.001, *n* = 116) as compared to normal breast tissues (*n* = 94).

CAPER protein expression was assessed by immunohistochemistry within 192 ER+, 48 HER2+ and 116 TN breast cancers compared to 94 normal human breast tissue samples (Figure 1A). For this purpose, tissue microarrays obtained from US Biomax Inc. were immunostained with CAPER antibody and expression levels were quantified by the proportion of positively-stained nuclei using the histoscore (H-score) method. Interestingly, our results revealed a significant increase of CAPER expression in all major breast cancer subtypes when compared to normal healthy breast specimens (Figure 1B; ER+; 2.5-fold, *p <* 0.001, *n* = 192; HER2+; 1.7-fold, *p <* 0.05, *n* = 48; TNBC; 2-fold, *p <* 0.001, *n* = 116). Although there was an increase in CAPER expression in all breast cancer subtypes, we focused on TNBC as this subtype is in the most need of targeted therapy.

### Upregulation of CAPER expression in a human TNBC cell line panel

**Figure 2:**
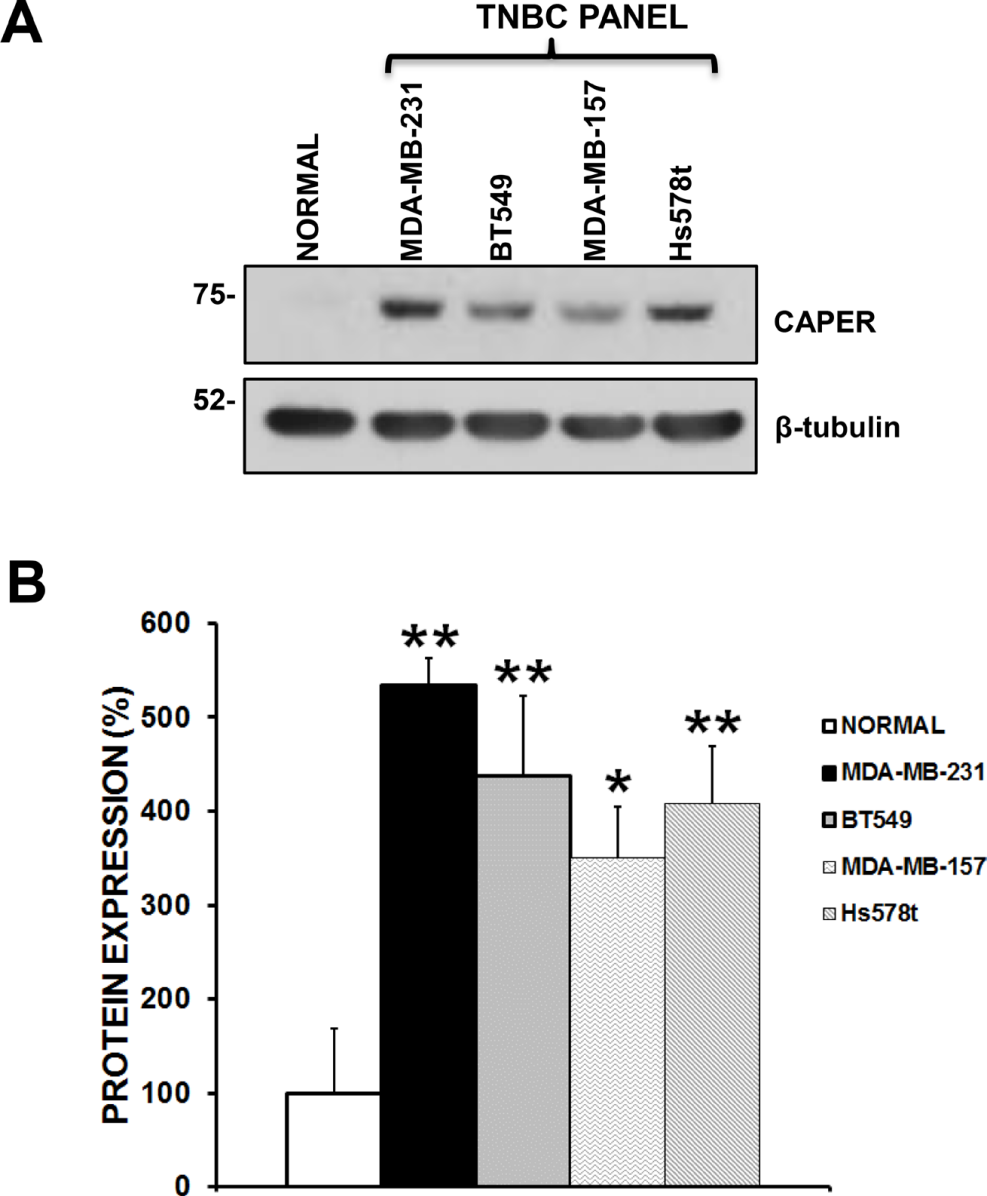
Endogenous CAPER levels in a panel of human tnbc cell lines (**A**) Western blot analysis demonstrates that CAPER protein levels are upregulated in a panel of TNBC cell lines (MDA-231, BT549, MDA-MB-157, Hs578t) as compared to normal primary breast epithelial cells (PCS-600-010). β-tubulin is shown as a control for equal loading. (**B**) As assessed by densitometry using Image J, TNBC cell lines show a significant increase in CAPER protein expression when compared to normal cells (BT549; 4.3-fold, *p <* 0.01, *n* = 3; MDA-MB-231; 5.3-fold, *p <* 0.01, *n* = 3; MDA-MB-157; 3.5-fold, *p <* 0.05 *n* = 3; Hs578t; 4-fold, *p <* 0.01, *n* = 3).

To begin understanding the functional role of CAPER in TNBC growth, we first determined its expression levels in a panel of 4 human TNBC cell lines (MDA-MB-231, BT549, MDA-MB-157 and Hs578t) as compared to normal primary human mammary epithelial cells (Figure 2A). Quantitatively, we confirmed that CAPER expression was significantly higher in TNBC cells than normal mammary epithelial cells (Figure 2B; MDA-MB-231; 5.3-fold, *p <* 0.01, *n* = 3; BT549; 4.3-fold, *p <* 0.01, *n* = 3; MDA-MB-157; 3.5-fold, *p <* 0.05, *n* = 3; Hs578t; 4-fold, *p <* 0.01, *n* = 3). These data suggest that human TNBC cells overexpress CAPER compared to normal primary human mammary epithelial cells, although its functional role remains elusive.

### Knockdown of CAPER expression prevents the growth of TNBC cells

**Figure 3:**
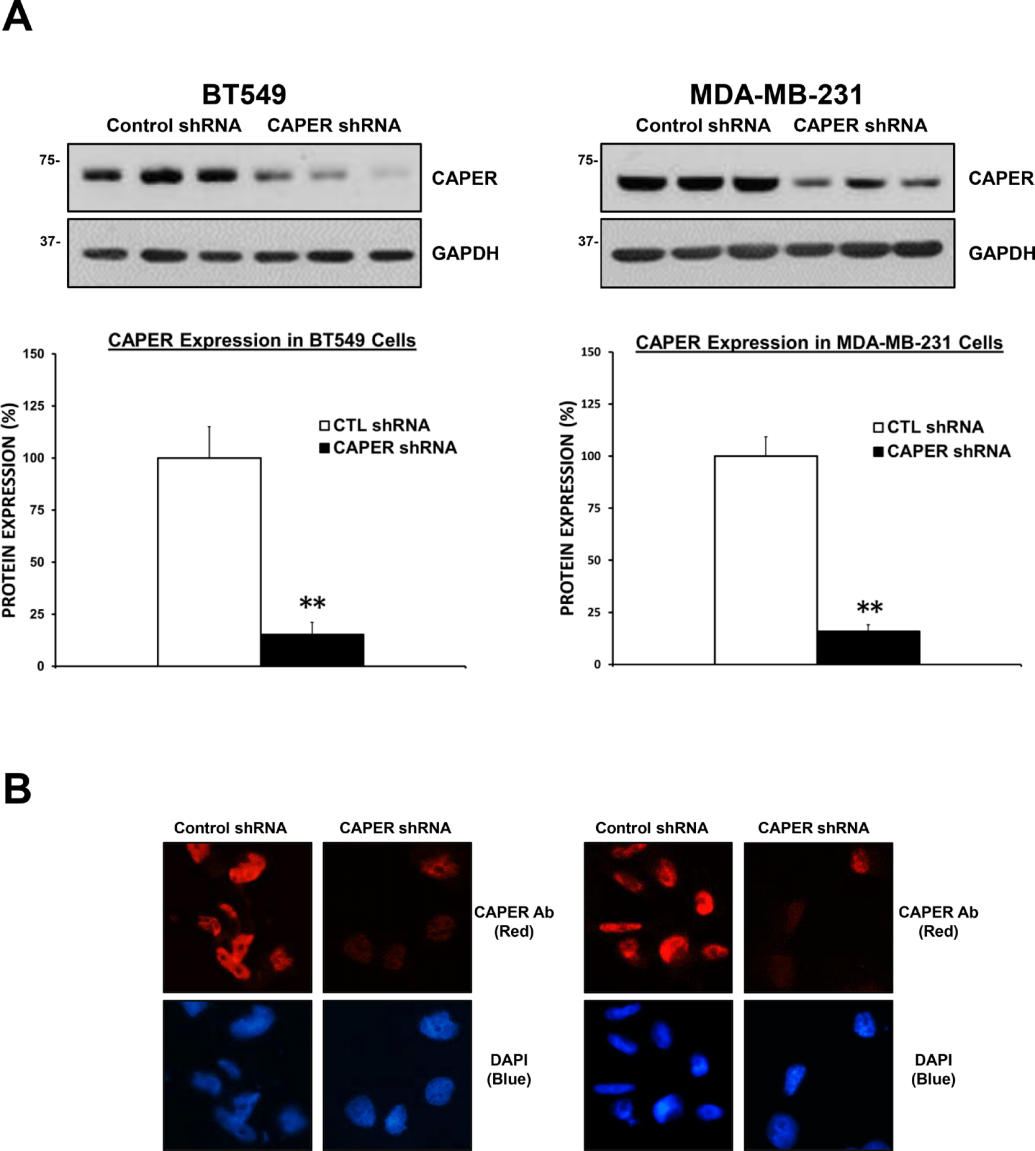
Validation of lentiviral-mediated knockdown of CAPER protein expression in tnbc cell lines (**A**) Western blot analysis shows reduced CAPER protein expression levels in BT549 and MDA-MB-231 cells expressing CAPER shRNAs compared to CTL shRNAs shRNAs. GAPDH is shown as loading control. Quantitation of CAPER knockdown was performed through densitometry using Image J and revealed a significant downregulation of CAPER protein levels in both cell lines (BT549 6.6-fold, *p <* 0.01, *n* = 3; MDA-MB-231 6.3-fold, *p <* 0.01, *n* = 3). (**B**) Qualitatively, CAPER protein expression was significantly reduced following CAPER shRNA knockdown in BT549 and MDA-MB-231 cells as assessed by immunofluorescence using EVOS-FL fluorescent microscope (40× objective). As seen CAPER expression was restricted to the nucleus (upper left panel) and significantly decreased following CAPER shRNA (right upper panel). As seen in bottom left and right panels, nuclear counterstain with DAPI illustrates nuclear localization in MDA-MB-231 cells (red; CAPER immunostaining, blue; nuclear DAPI staining).

**Figure 4:**
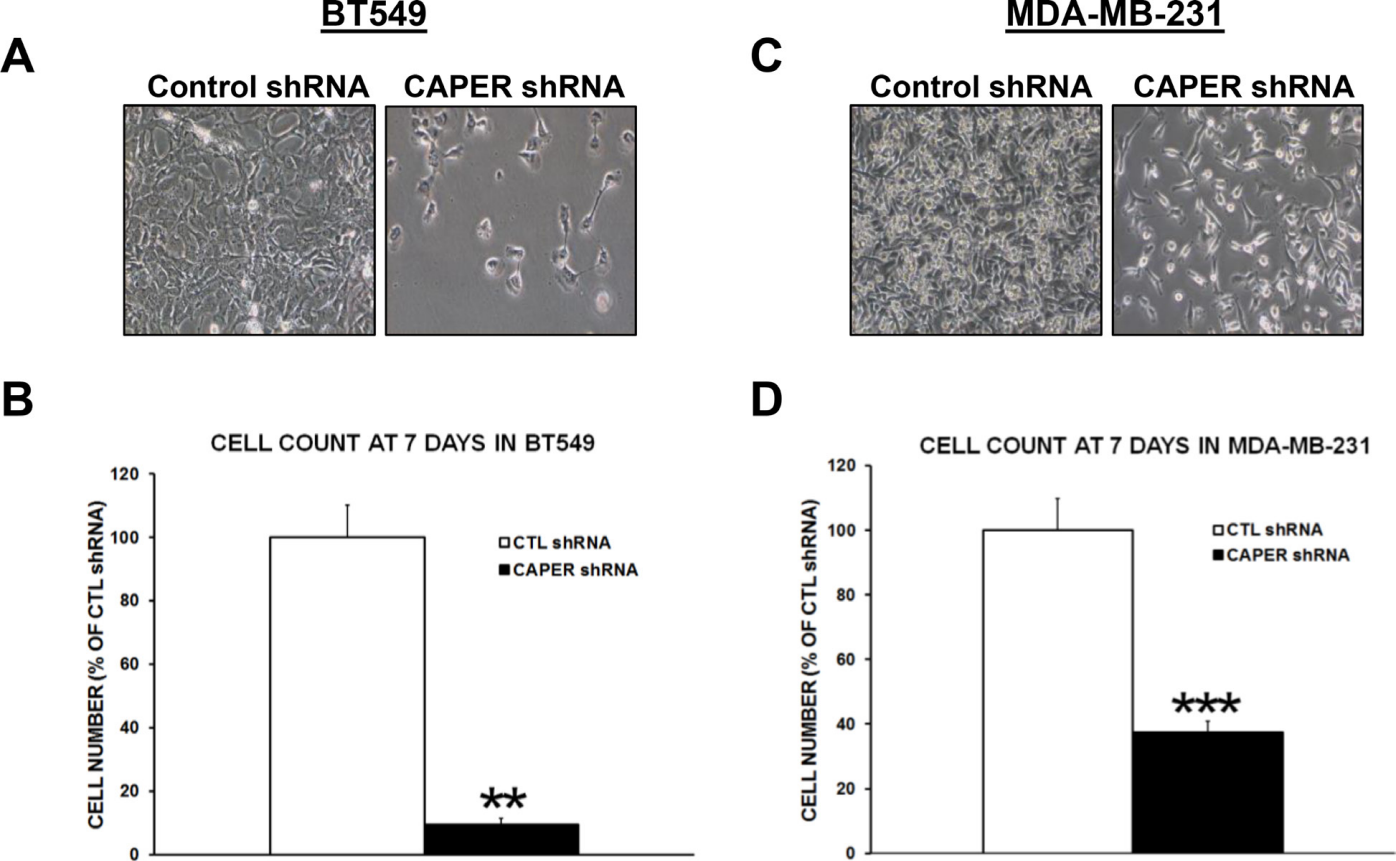
Knockdown of CAPER expression reduces number of adherent TNBC cells *in vitro* Phase contrast pictures depict a visual difference in adherent cell numbers at 7-day time point following equal plating in (**A**) BT549 and (**C**) MDA-MB-231 cells expressing either control or CAPER shRNAs (pictures acquired using a 10× objective, Olympus). Quantitatively, total cell count of adherent cells clearly demonstrates that knockdown of CAPER significantly decreases cell number in (**B**) BT549 (~10-fold, *p <* 0.01, *n* = 4) and (**D**) MDA-MB-231 cells (~2.5-fold, *p <* 0.001, *n* = 8) vs CTL shRNA.

To determine the functional role of CAPER in human TNBC pathogenesis, we used a lentiviral-mediated gene silencing approach to reduce the expression of CAPER in human TNBC cell lines. For this purpose, two different cell lines expressing endogenous CAPER protein levels were selected. CAPER shRNA BT549 and MDA-MB-231 cells demonstrated a significant decrease in CAPER protein levels (BT549; 6.6-fold, *p <* 0.01, *n* = 3; MDA-MB-231; 6.3-fold, *p <* 0.01, *n* = 3) compared to CTL shRNA (Figure 3A). In addition, qualitative analysis of CAPER expression revealed a decrease in the levels of nuclear CAPER in CAPER shRNA cells as assessed by immunofluorescence when compared to CTL shRNA counterparts (Figure 3B). As shown in Figure 4A and 4C, when seeded equally, cells expressing CAPER shRNAs were visually less confluent than their CTL shRNA equivalents following a 7-day culture. Quantitatively, knockdown of CAPER expression in TNBC cells significantly decreased total cell numbers in BT549 (10-fold, *p <* 0.01, *n* = 4) and MDA-MB-231 cells lines (2.5-fold, *p <* 0.001, *n* = 8) vs CTL shRNA (Figure 4B and 4D).

### Confirming growth inhibitory effect of CAPER using a different shRNA sequence

**Figure 5:**
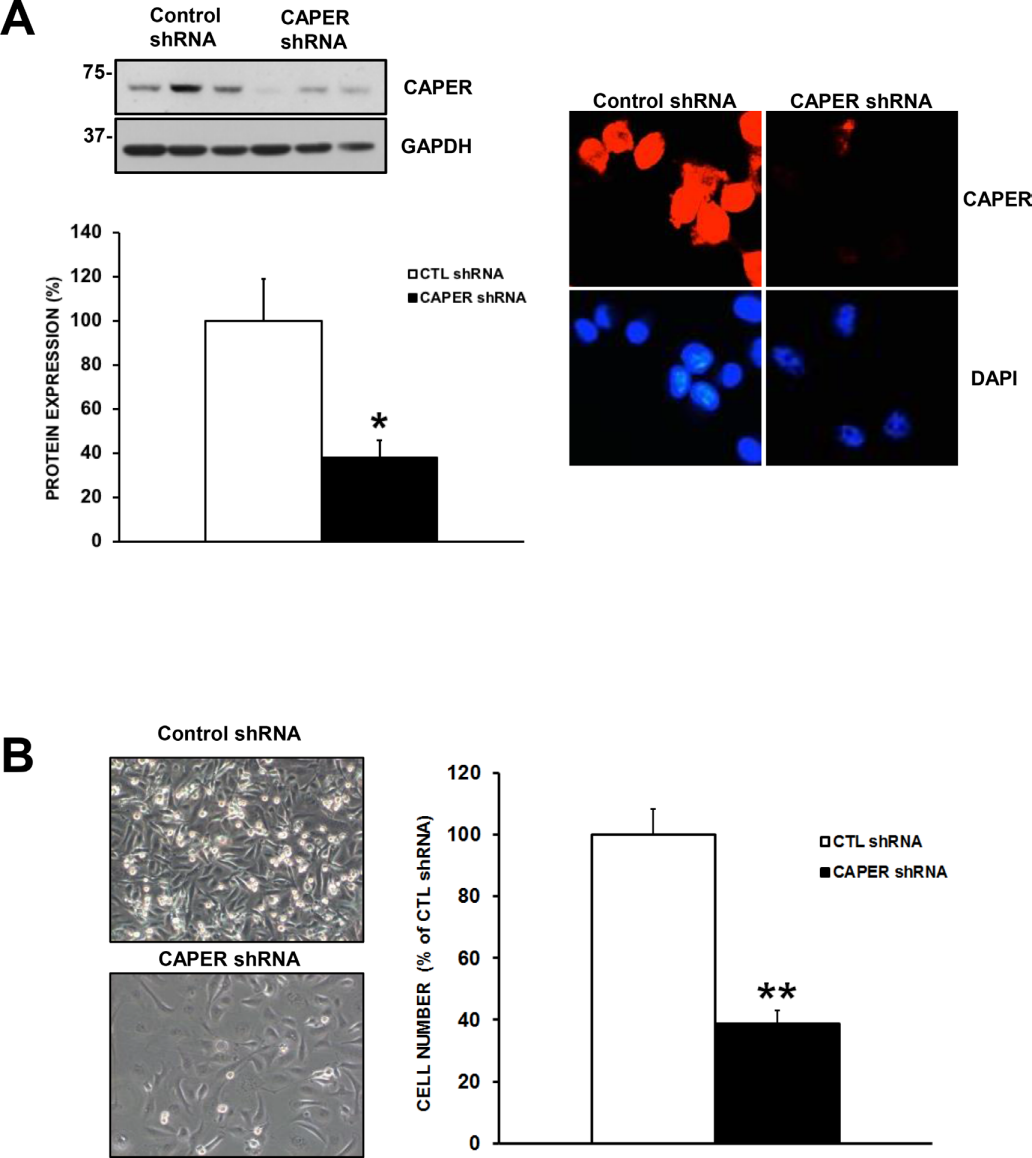
Knockdown of CAPER expression with a different shRNA sequence also diminishes cell number (**A**) Western blot analysis demonstrates that MDA-MB-231 cells stably expressing CAPER shRNA #69 (TRCN0000021769) also demonstrate reduced CAPER protein expression. Indeed, quantitation of CAPER knockdown through densitometry using Image J (CAPER/GAPDH) revealed a significant downregulation of CAPER protein levels (~2.5-fold, *p <* 0.05, *n* = 3) vs CTL shRNA. As seen CAPER expression was restricted to the nucleus (upper left panel) and significantly decreased following CAPER shRNA (right upper panel). Nuclear counterstain with DAPI illustrates nuclear localization in MDA-MB-231 cells (red; CAPER immunostaining, blue; nuclear DAPI staining). Immunofluorescence pictures were acquired on EVOS-FL using a 40× objective. (**B**) Phase contrast pictures depict a visual difference at 7-day time point following equal plating in MDA-MB-231 expressing CAPER shRNAs (pictures acquired using a 10× objective, Olympus). Quantitatively, total cell count of adherent cells clearly demonstrates that knockdown of CAPER significantly reduced cell number (~2.5 fold, *p <* 0.01, *n* = 3) as compared to their control counterpart.

As shown in Figure 5A, Western blot and immunofluorescence analyses confirmed a consistent knockdown of CAPER protein levels in MDA-MB-231 cells harboring the CAPER shRNA #69 (TRCN0000021769) (2.5-fold, *p <* 0.05, *n* = 3) vs CTL shRNA. Furthermore, Figure 5B shows similar effects of shRNA #69 on MDA-MB-231 cell count (2.5-fold, *p <* 0.01, *n* = 3) vs CTL shRNA. As we now validated two different shRNA CAPER sequences, we pursued our mechanistic studies with shRNA #70.

### Knockdown of CAPER in a non-tumorigenic cell line MCF-10A does not affect their growth

**Figure 6:**
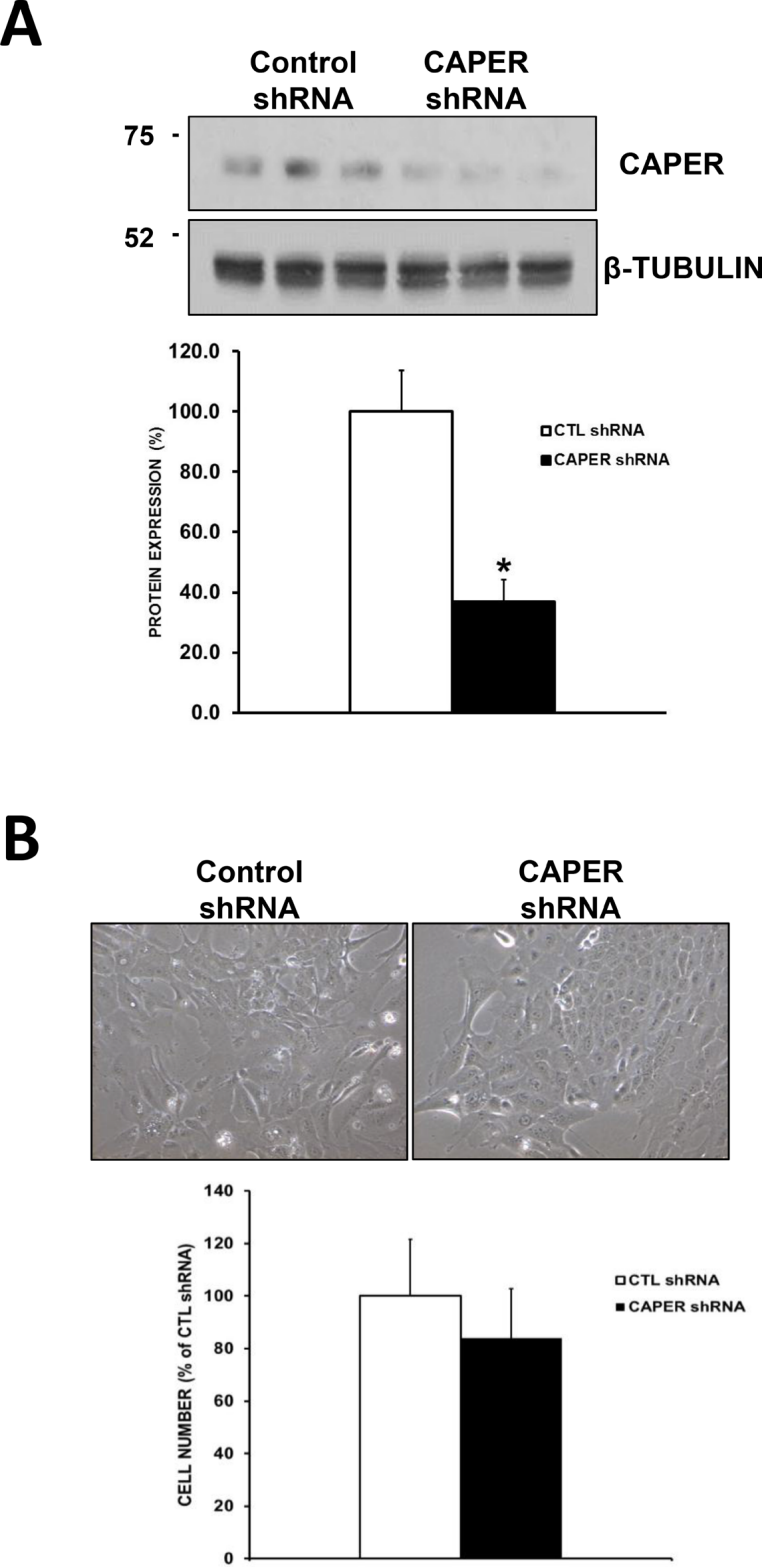
Knockdown of CAPER in a non-tumorigenic cell line MCF-10A does not affect their growth (**A**) Western blot analysis shows reduced CAPER protein expression levels in MCF-10A cells expressing CAPER shRNAs compared to CTL shRNAs. Beta-tubulin is shown as loading control. Quantitation of CAPER knockdown was performed through densitometry using Image J and revealed a significant downregulation of CAPER protein levels in MCF-10A cell line (2.7-fold, *p <* 0.05, *n* = 3). (**B**) Phase contrast pictures depict no visual difference in adherent cell numbers at 7-day time point following equal plating in MCF-10A cells expressing either CTL or CAPER shRNAs (pictures acquired using a 10× objective, Olympus). Quantitatively, total cell count of adherent cells clearly demonstrates that knockdown of CAPER did not significantly affect cell number in MCF-10A (*p* = NS, *n* = 4) vs CTL shRNA.

Using a lentiviral-mediated gene silencing approach as described above, we reduce the expression of CAPER in the human non-tumorigenic cell line MCF-10A. While CAPER shRNA caused a significant decrease in CAPER protein levels (2.7-fold, *p <* 0.05, *n* = 3) compared to CTL shRNA (Figure 6A), when seeded equally, cells expressing CAPER shRNAs did not grow significantly different than their CTL shRNA equivalents following a 7-day culture (Figure 6B, *p* = NS, *n* = 4).

### Knockdown of CAPER in MDA-MB-231 cells leads to activation of DNA damage proteins γH2AX (ser139) and phospho-ATM (ser1981) and promotes apoptosis without affecting cell cycle

**Figure 7:**
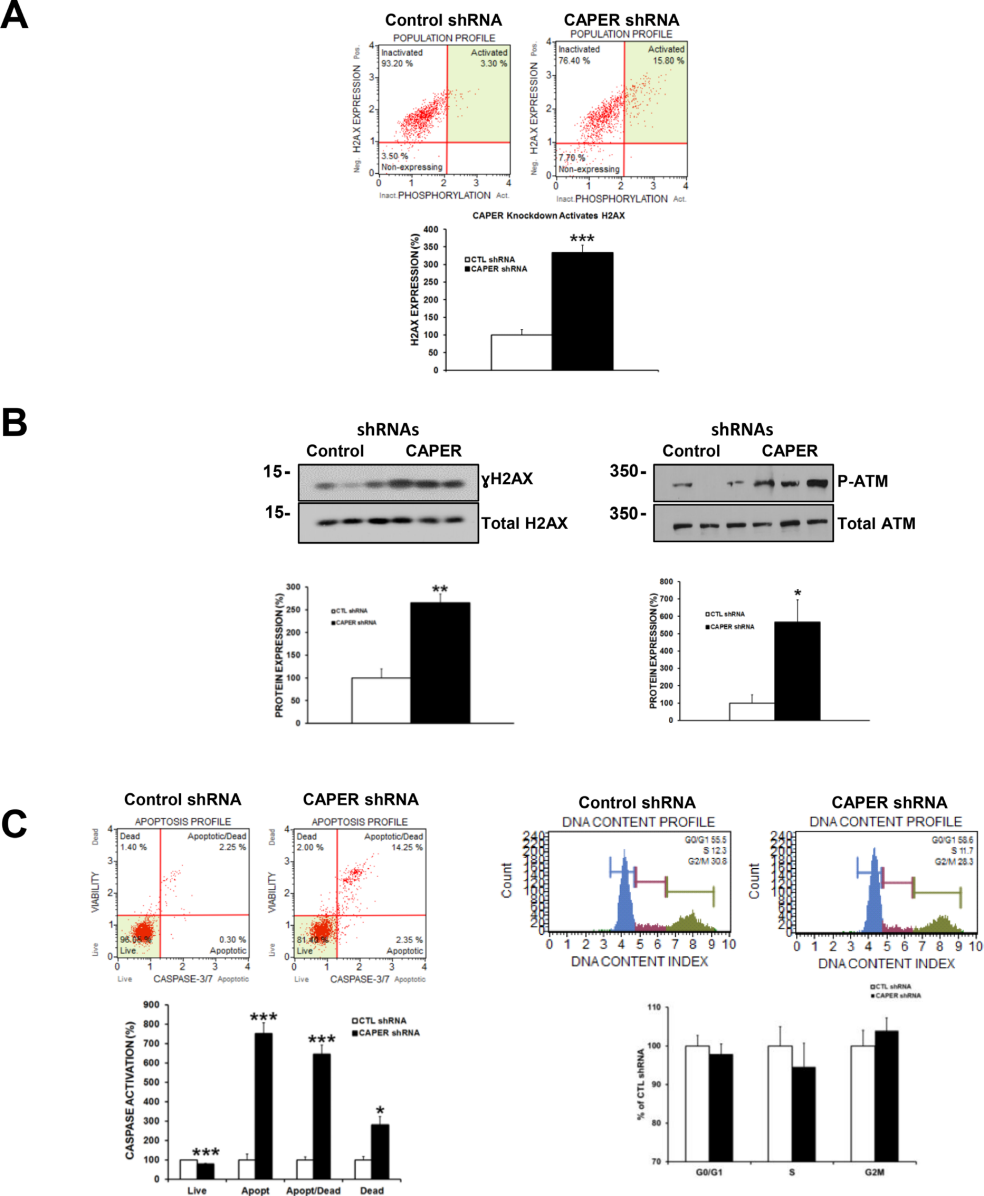
Knockdown of CAPER induces DSB proteins ATM and H2AX and leads to apoptosis in MDA-MB-231 cells (**A**) H2AX phosphorylation on ser139 is significantly increased (upper right quadrant population) in MDA-MB-231 cells after CAPER knockdown as represented by Muse Cell Analyzer plots (3-fold, *p <* 0.001, *n* = 3). (**B**) The increase in γH2AX was also validated through western blot analysis (2.5-fold, *p <* 0.01, *n* = 3). ATM phosphorylation on serine1981 is significantly upregulated after knockdown of CAPER expression (5-fold, *p <* 0.05, *n* = 3). (**C**) CAPER knockdown resulted in an increased level of caspase-3/7 activation through decreasing live cells (1.2-fold, *p <* 0.001, *n* = 3), while increasing apoptotic (7.5-fold, *p <* 0.001, *n* = 3), apoptotic/dead (6.5-fold, *p <* 0.001, *n* = 3), and dead (3-fold, *p <* 0.05, *n* = 3) cell populations. Interestingly, MDA-MB-231 cells expressing CAPER knockdown displayed no significant changes in any of the phases of the cell cycle (*p* = NS, *n* = 8, for G1, S and G2/M phases) compared to CTL shRNA (Figure 7C right panels).

The decrease in cell number observed in CAPER knockdowns could be a result of increased cell death. A recent study recently attributed a novel role of CAPER in DNA damage response [12], a pathway often resulting in increased cell death when misregulated. To further uncover the mechanistic role of CAPER in our TNBC model, we began investigating how CAPER knockdown regulates DNA damage response pathways. Interestingly, as shown by a Muse assay, CAPER knockdown induced the phosphorylation of H2AX (γH2AX) on serine 139 (upper right quadrant population) suggesting more DNA damage (breaks) is occurring in these cells (Figure 7A; 3-fold, *p <* 0.001, *n* = 3). This increase in γH2AX was also validated through Western blot analyses (Figure 7B, left panel; 2.7-fold, *p <* 0.01, *n* = 3). In addition, CAPER knockdown also significantly upregulated the phosphorylation of ATM on ser1981, another protein involved in signaling the presence of DNA damage (Figure 7B, right panel; 5-fold, *p <* 0.05, *n* = 3). Moreover, MDA-MB-231 cells expressing CAPER knockdown displayed a significant decrease in live cells (1.2-fold, *p <* 0.001, *n* = 3), an increase in early apoptotic cells population (7.5-fold, *p <* 0.001, *n* = 3), apoptotic/dead cells population (6.5-fold, *p <* 0.001, *n* = 3), and dead cells population (3-fold, *p <* 0.05, *n* = 3) as assessed by a caspase-3/7 Muse apoptosis assay (Figure 7C, left panels). Interestingly, MDA-MB-231 cells expressing CAPER knockdown displayed no significant changes in any of the phases of the cell cycle (*p* = NS*, n* = 8, for G1, S and G2/M phases) compared to CTL shRNA shRNA (Figure 7C, right panels).

### CAPER knockdown decreases the levels of c-Abl, RAD51 and retinoblastoma proteins, essential proteins involved in homologous recombination repair of DNA in MDA-MB-231 cells

**Figure 8:**
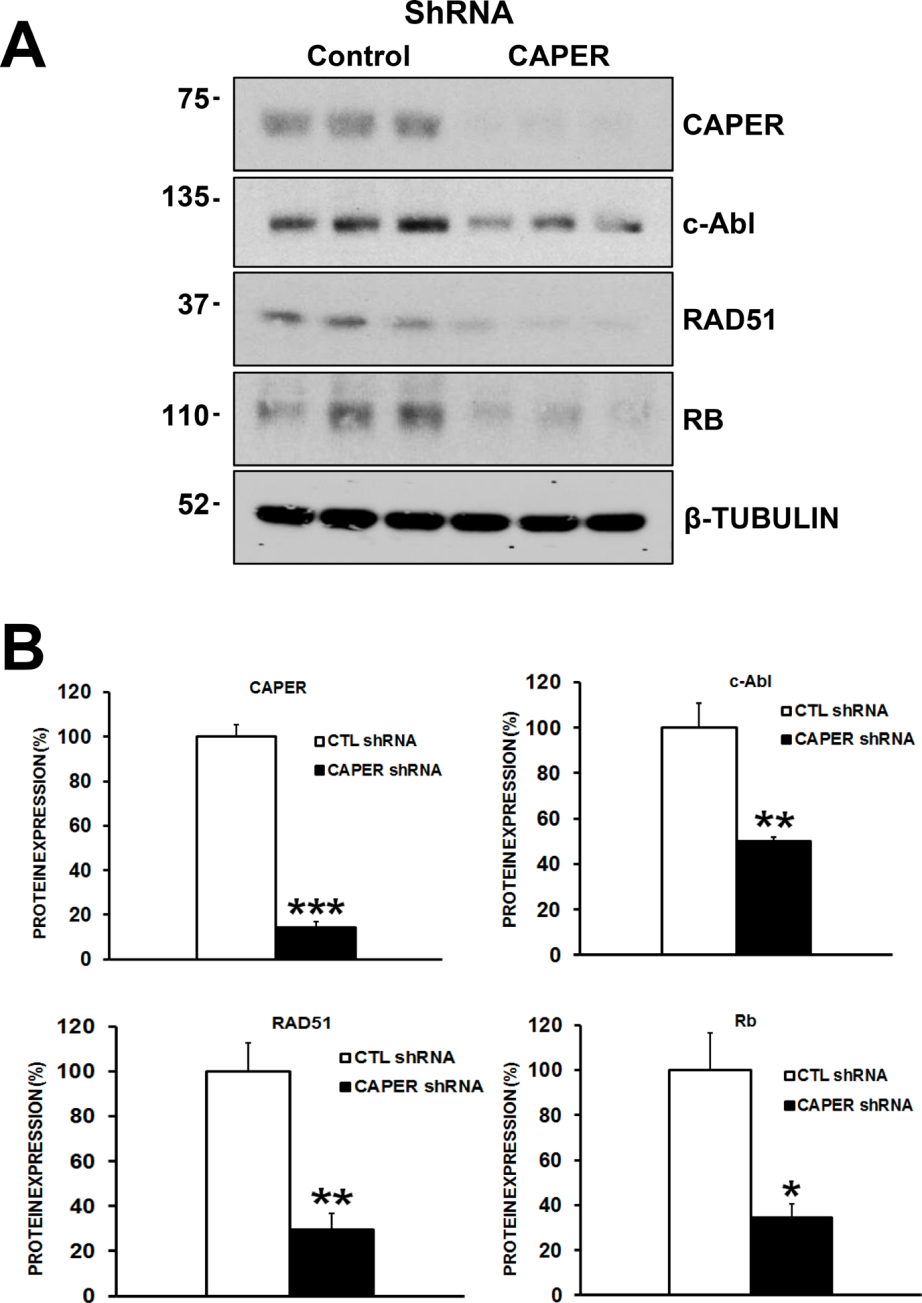
CAPER Knockdown decreases the levels of c-Abl, RAD51 and retinoblastoma proteins, essential proteins involved in homologous recombination repair of DNA in MDA-MB-231 cells (**A**) Western blot analysis shows the effects of CAPER knockdown on DNA repair protein expression levels. (**B**) Quantitation was performed through densitometry using Image J which revealed that down-regulation of CAPER (7-fold, *p <* 0.001, *n* = 3) following lentiviral infection caused significant changes in c-Abl (*p <* 0.01, 2-fold, *n* = 3), RAD51 (*p <* 0.01, 2.5-fold, *n* = 3) and Rb (*p <* 0.05, 3-fold, *n* = 3) protein levels in MDA-MB-231 cells.

Interestingly, the expression of important DNA repair proteins was significantly decreased upon CAPER knockdown in MDA-MB-231 cells. In fact, following Western blot analyses, we observed significant decreases in the total protein levels of c-Abl (2-fold, *p <* 0.01, *n* = 3), retinoblastoma protein (Rb) (3-fold, *p <* 0.05, *n* = 3) and RAD51 (3.5-fold, *p <* 0.01, *n* = 3), suggesting CAPER's involvement in regulating effector DNA repair proteins (Figure 8A and 8B).

## DISCUSSION

Although previously shown to be upregulated in invasive breast cancer and DCIS, CAPER expression has never been reported among specific histological subtypes. Our present data demonstrate for the first time an upregulation of CAPER protein expression in a cohort of patients as part of each major breast cancer subtypes (ER+, HER2+, and TNBC) compared to normal breast tissue as defined by H-scoring method [13]. These immunohistochemical results emphasize that CAPER expression might be needed for the growth of several different breast cancer subtypes. In fact, our group previously showed that knockdown of CAPER in an ER-dependent cell line significantly impaired their growth both *in vitro* and *in vivo* [12]. Since TNBC possesses the most aggressive features, we focused the current study on the functional role of CAPER in that subtype. However, it remains to be confirmed whether CAPER knockdown could also prevent the growth of HER2+ cancer cells and this should be investigated further in future studies.

Our current results demonstrate that lentiviral-mediated knockdown of CAPER expression reduced total adherent cell number in TNBC cell lines MDA-MB-231 and BT549, an effect attributed to increased apoptotic cell death through caspase-3/7 activation. These results suggest that CAPER could be involved in hormone-independent functions. This is a departure from its original role predicted to be mostly ER-dependent [9]. Other studies have also indicated a hormonal-independent role of CAPER in other cancers such as colon cancer in which CAPER knockdown also pushed the cells towards apoptosis-mediated cell death [14, 15]. Our current data, in addition to other published studies, validate that CAPER is involved in several different cancer types and does not necessarily require hormonal-dependence to exhibit a significant effect on cancer growth. These observations make CAPER a very attractive therapeutic target for cancer treatment. This is consistent with other proteins shown to also be involved in ER-independent processes following their discovery as estrogen-receptor coactivators. The ER coactivator SRC-3 has been shown to correlate with poor prognosis and overall survival in TNBC and its inhibition was shown to reduce tumor growth in an *in vivo* TNBC model [16]. Proline-, glutamic acid-, and leucine-rich protein 1 (PELP-1), another ER coactivator, has also been implicated in the survival and metastatic potential of TNBC cells [17]. Knockdown of PELP-1 was shown to reduce the proliferation and migration of TNBC cells [17]. Together, these results support the involvement of other ER coactivators in hormone-independent cancers.

Mechanistically, our current results show that CAPER knockdown induces an apoptotic response in TNBC cells through caspases activation while consistently failing to show any effect on cell cycle. This contrasts with our previous study in MCF-7 cells where S-phase proteins such as proliferating cell nuclear antigen (PCNA) decreased following CAPER knockdown, suggesting that growth-inhibition linked to cell cycle regulation could be hormonal-dependent [12]. These observations are in accordance with Sillars-Hardebol *et al*. who showed that CAPER knockdown in colon cancer cells reduced their cell viability [15]. This could suggest that CAPER possesses different functions on cell growth whether its cancer cell host is hormonal-dependent or independent. This could be due to the regulation of AP-1 by CAPER in different subtypes of breast cancer. In our previous studies, we observed that CAPER knockdown induced a decrease in c-jun in MCF-7 cells while the levels remained unchanged in TNBC (data not shown). AP-1 has previously been linked to cell cycle regulation such as regulating the levels of PCNA [18]. The lack of cell cycle effects observed following CAPER knockdown in TNBC cells might be due to a lack of effect on the AP-1 pathway in a hormonal-independent context and will remain to be further investigated.

We thus wanted to further delineate the underlying mechanisms that might be responsible for the induction of apoptosis following CAPER downregulation in TNBC cells. Apoptosis is a common cellular response aiming to protect organisms against irreparable DNA damage [19, 20]. Interestingly, CAPER has recently been implicated in the regulation of DNA repair related genes through alternative splicing as detected by RNA sequencing [10]. We therefore focused on furthering our mechanistic knowledge on the specific role of CAPER in DNA damage/repair response. We hypothesized that CAPER actively contributes to maintaining functional DNA repair in TNBC cells and when CAPER is downregulated, more DNA damage will result due to an inefficient repair. We first looked at ATM (ataxia-telangiectasia mutated), a kinase in the phosphoinositide 3-kinase-related protein kinase (PIKKs) family activated in the vicinity of a double strand break (DSB) through autophosphorylation on serine 1981 [21]. Phosphorylation of ATM on this residue is a hallmark of DNA damage and CAPER knockdown increased its levels significantly, signaling the presence of DSBs. In addition, CAPER knockdown also increased phospho-H2AX (serine 139; Ɣ -H2AX) expression, another early event in the DNA repair response to DSBs, a modified histone protein regulated by ATM. This further validated the presence of DSBs in MDA-MB-231 cells following CAPER knockdown.

We then sought to understand why TNBC cells lacking CAPER expression are incapable of efficiently repairing these DSBs. DNA can be repaired through homologous recombination (HR) in which RAD51 is a central protein. RAD51 knockdown cells have shown increased Ɣ -H2AX foci accumulation after treatment with temozolomide, suggesting that disrupting RAD51 protein expression promotes defective HR leading to chemotherapy-induced cell death [22]. Here, we show a significant decrease in total protein levels of RAD51 after knockdown of CAPER in MDA-MB-231 cells, suggesting CAPER crosstalks with essential repair proteins involved in DSB repair. Clinically, this could be very important as low RAD51 levels have been associated with favorable response after chemotherapy in breast cancer patients, due to the lack of repair following DNA damaging therapies [23]. Thus clinically, it remains to be determined if CAPER levels following chemo- or radiation therapy could also serve as a predictive marker of therapeutic response in TNBC patients and whether RAD51 decrease following a successful response to therapy is directly regulated by CAPER. We postulate that the effects of reduced RAD51 expression by CAPER knockdown causes a constitutive activation of ATM and H2AX which are attempting to signal sustained DNA damage and attempting to reactivate RAD51. Furthermore, the induction of RAD51 repair protein complex is dependent on the interaction between ATM and non-receptor tyrosine kinase c-Abl [24, 25]. c-Abl is involved in various cell processes including DNA repair and apoptosis [26]. c-Abl and RAD51 can be immunoprecipitated together, suggesting their physical interaction [24]. Recent data has also shown a direct interaction between CAPER and c-Abl, suggesting a possible link between CAPER, c-Abl, and RAD51 [27]. In addition, retinoblastoma (Rb) protein has been shown to bind directly to c-Abl and is a key factor in DNA repair and protecting cells from apoptosis [28–30]. Our results also showed a decrease in total protein levels of c-Abl and Rb in addition to RAD51 as a direct result of decreasing total CAPER protein levels in MDA-MB-231 cells.

Downregulation of these important DNA repair proteins could be happening at different levels following CAPER knockdown. This could be due to a direct or indirect effect of CAPER on their protein stability, mRNA processing, gene transcription or the direct involvement of CAPER in the splicing of these genes. C-Abl, RAD-51 and Rb should be studied individually to test these different hypotheses. Another possibility could be that c-Abl, RAD51, Rb and CAPER are part of the same complex and that a downregulation of CAPER alters the stability of these interactions leading to degradation. This latter possibility could suggest that CAPER is essential in stabilizing the expression of these effector repair proteins in TNBC cells and that their misregulation by CAPER downregulation could lead to unsuccessful DNA repair and apoptotic cell death. In addition, it would be interesting to knockdown these different DNA repair proteins in TNBC cells and observe if this would recapitulate the effects seen following CAPER knockdown. The cellular mechanisms by which CAPER decreases protein levels of c-Abl, RAD51 and Rb and how these interact with each other and with CAPER during DNA repair remains to be elucidated.

A major roadblock in current chemotherapies available for TNBC patients is the accompanied toxicity attacking normal cells in the body due to lack of selectivity. A major effort is to currently explore targeted therapies that take advantage of DNA repair pathways that are selectively upregulated in cancer cells to fight DNA damage. In the current study, CAPER knockdown in a non-tumorigenic cell line (MCF-10A) did not significantly alter their growth, suggesting a potentially selective effect of CAPER targeting on cancer cells. Previous work demonstrated that clinical specimens from bladder, breast, lung and colon tumors, but not normal tissues, commonly express markers of an activated DNA damage response [31]. We thus hypothesize that TNBC cells might be more reliant on CAPER expression and DNA repair than MCF-10A cells, making them more susceptible to cell death following CAPER downmodulation. More studies will be required to elucidate the activity of CAPER with regards to DNA damage in TNBC vs normal cell lines. Interestingly, several studies have shown increased efficiency in DNA repair pathways within cancer cells might contribute to their aggressive behaviors and resistance to therapy. For example, RAD51 has been linked to resistance to PARP inhibition in TNBC and its inhibition was shown to sensitize these cells to olaparib both *in vitro* and *in vivo* [22, 32, 33]. As such targeting CAPER levels in TNBC, alone or in combination with DNA-damaging agents, could be an interesting new therapeutic avenue for the treatment of TNBC. This further illustrates how important it is to understand DNA repair proteins pathways in TNBC. Although other targeted therapies such as inhibitors of the androgen receptor [7, 34], PARP [35, 36], FGFR [7] and gamma secretase [7, 37] are currently being sought for the treatment of TNBC, there is room for improvement and more still needs to be discovered about DNA damage targets within TNBC cells.

In summary, our current study provides the first evidence of CAPER's role in TNBC and its key function in cell survival through DNA repair pathways. Our data suggest that CAPER-deprived TNBC cells could undergo more DNA damage through malfunctions in effector DNA repair proteins c-Abl, RAD51 and Rb. Failure of these important DNA repair pathways during CAPER deprivation in TNBC leads to apoptosis and death. CAPER could become an important biomarker in TNBC to predict response to DNA damaging therapies and allow for more efficient cell death when targeted in aggressive TNBC.

## MATERIALS AND METHODS

### Materials

This study was conducted according to the guidelines of the National Institutes of Health (NIH) and the USciences’ Institutional Biosafety Committee (IBC). MDA-MB-231 (cat# HTB-226), BT549 (cat# HTB-122), MDA-MB-157 (cat# HTB-24), Hs578T (cat# HTB-126), normal primary mammary epithelial cells (cat# PCS-600-010) and MCF-10A (cat# CRL-10317) were purchased from American Type Culture Collection (ATCC, Manassas, VA). Transduction-ready control shRNA (cat# SHC001V) and human CAPER shRNA lentiviral particles (cat# SHCLNV-NM_004902 (clone# TRCN0000021770 and TRCN0000021769 abbreviated as shRNA #70 and #69)) were purchased from Sigma-Aldrich (St. Louis, MO). Rabbit polyclonal anti-CAPER antibody (cat# 3733-100) was purchased from BioVision Inc (Milpitas, CA). Primary antibodies against Ɣ -H2AX (cat# 9718), c-Abl (cat# 2862), RAD51 (cat# 8875), and Rb (cat# 9309) were purchased from Cell Signaling Technology (Danvers, MA). Phospho-ATM antibody (cat# ab81292) and total ATM (cat#ab32420) were purchased from Abcam (Cambridge, MA). Total H2AX (cat#100638) was purchased from Novus Biologicals (Littleton, CO). Rabbit polyclonal anti-β-tubulin antibody (cat# ab6046) was purchased from Abcam (Cambridge, MA). A mouse mAb to glyceraldehyde 3-phosphate dehydrogenase (GAPDH, cat# 10R-G109a) was purchased from Fitzgerald Industries (Acton, MA).

### Human specimens

Tissue microarrays were commercially obtained from US Biomax (Rockville, MD) (cat# BR487a, BRN801a, BR1921a, BR1503, HBre-Dub090Sur-01 and HBre-Duc150Sur-01). Data from these tissue microarrays stained with CAPER antibody was compiled from 116 women diagnosed with TNBC, 192 women with ER+, and 48 women with HER2+ breast cancers and compared to 94 breast tissue samples from healthy women.

### Immunohistochemistry

Paraffin-embedded microarrays were dehydrated in xylene for 1 hr and rehydrated in a series of graded ethanol and completely rehydrated in double distilled water for 5 minutes. Antigen retrieval was performed using 0.1M citric acid and 0.1M sodium citrate for 5 minutes in a pressure cooker and cooled to room temperature. Slides were washed with 1X PBS and incubated for 30 minutes with 3% hydrogen peroxide, then washed and blocked for 1 hr at room temperature with 10% normal goat serum (cat# S-1000, Vector Labs, Burlingame, CA) in 1X PBS. Slides were incubated with rabbit polyclonal anti-CAPER primary antibody (cat# 3733-100, Biovision) (1:50) overnight at 4° C. Slides were washed with 1X PBS and blocked with streptavidin/biotin blocking kit (cat# SP-2002, Vector Labs) at room temperature for a total of 20 minutes as per manufacturer's instructions. After washing in 1X PBS, slides were incubated with rabbit biotinylated secondary antibody (1:250) (cat# BA-1000, Vector Labs) at room temperature for 30 minutes, washed in 1X PBS, and incubated with streptavidin-HRP tertiary antibody (cat# P0397, Dako, Santa Clara, CA) (1:500) for 30 minutes. Slides were then washed with 1X PBS and incubated with 3,3-diaminobenzidine (DAB) substrate (cat# K3468, Dako) for 15 seconds. Slides were counterstained with Mayer hematoxylin (cat# TA-125-MH, Fisher Scientific, Waltham, MA) for 2 minutes, dehydrated, and mounted with Permount (cat# SP15100, Fisher Scientific).

### Immunohistochemical scoring of tissues

Since CAPER is active when expressed in the nucleus, the proportion of cells positive for nuclear CAPER (following immunohistochemical staining with CAPER antibody) was assessed and quantitated by board-certified pathologists at Cooper University Hospital, Camden, NJ [13]. The intensity of nuclear staining was scored for individual tumor cell nuclei as negative (–)/no staining, staining weakly (1+), staining intermediately (2+), or staining strongly (3+). A minimum of 100 tumor cells were scored with the percentage of tumor cell nuclei in each category recorded. A semi-quantitative histology score (H-score) was assigned to tumor samples as follow: H-score = [1 × (% cells 1+) + 2 × (% cells 2+) + 3 × (% cells 3+)]. The final score, ranging from 0–300, gives more accurate score intensity staining in a given tumor sample. The sample can then be considered positive or negative on the basis of a specific discriminatory threshold.

### Lentiviral infection of MDA-MB-231 and BT549 cells

MDA-MB-231 cells obtained from ATCC were cultured in Dulbecco minimum essential medium (DMEM, cat# 11965, Gibco/Life Technologies, Carlsbad, CA) containing 10% fetal bovine serum (FBS cat# 16140, Gibco/Life Technologies), 1% penicillin/streptomycin (cat# 15140, Gibco/Life Technologies) and 1% sodium pyruvate (cat# 11360-070, Gibco/Life Technologies). BT549 cells were cultured in RPMI 1640 medium (cat# A10491, Gibco/Life Technologies) containing 10% FBS, 1% penicillin/streptomycin, and 0.023 IU/ml insulin (cat# I0516, Sigma-Aldrich). MCF-10A cells were cultured in DMEM/F12-K medium (cat# Gibco/Life Technologies) containing 5% horse serum (cat#30204, ATCC), 1% penicillin/streptomycin, 0.5 mg/ml hydrocortisone (cat#H0888, Sigma-Aldrich), 10 ug/ml insulin, 20 ng/ml EGF (cat#AF-100-15, Peprotech, Rocky Hill, NJ), and 100 ng/ml cholera toxin (cat#c8052, Sigma-Aldrich). When cells reached 50% confluency, complete medium was replaced with polybrene-containing medium (5 ug/ml, cat# sc-134220, Santa Cruz Biotechnology, Dallas, TX). MDA-MB-231, BT549 and MCF-10A cells were infected with 50 ul of transduction-ready control shRNA or human CAPER shRNA lentiviral particles in 5 mL of media for 24 hours. Stable cell lines were selected with puromycin dihydrochloride (1–2.5 ug/ml, cat# sc-108071, Santa Cruz Biotechnology) for 72 hours and grown for experimental purposes.

### Cell count

MDA-MB-231, BT549 and MCF-10A cells expressing either control (CTL) or CAPER shRNAs were seeded at a density of 200,000 cells per 10 cm culture dish (cat# 877223, Fisher Scientific) with media change at day 3. Seven days after initial plating, MDA-MB-231, BT549 and MCF-10A cells (CTL and CAPER shRNAs) cells were rinsed with 1X PBS (cat# 14190, Gibco/Life Technologies) and trypsinized with 0.05% trypsin-EDTA (cat# 25300, Gibco/Life Technologies) or 0.25% trypsin (cat# 15050, Gibco/Life Technologies), respectively. At day 7, adherent cells were counted and reported as a percentage of the control group (CTL shRNA).

### Western blot

Cells were washed twice with cold 1× PBS and collected in complete RIPA lysis buffer containing protease inhibitor cocktail (cat# 11-836-153-001, Roche, Basel, Switzerland) and Halt Phosphatase inhibitor single-use cocktail (cat# 78428, Fisher Scientific). The lysates were sonicated for 30 seconds on ice and centrifuged at 10,000 × g for 10 min at 4° C. The supernatants were then collected and protein concentration was determined using the bicinchoninic acid (BCA, cat# PI23250, Fisher Scientific) method. 50 ug of proteins were loaded in each lane and separated by sodium dodecyl sulfate polyacrylamide gel electrophoresis (SDS-PAGE) and transferred to nitrocellulose membranes. Membranes were incubated in blocking buffer for 1 hr then transferred to primary antibody incubation overnight at 4° C while shaking. Membranes were washed with 10 mM Tris, 150 mM NaCl, and 0.001% Tween 20 (1X-TBS-Tween) before addition of chosen HRP-conjugated secondary antibody (cat# 554021, BD Biosciences, San Jose, CA) for 1 hr. Super Signal chemiluminescent substrate (cat# sc-2048, Santa Cruz Biotechnology) was used for detection of HRP-conjugated antibody. Western blot images were quantified using Image J software analysis.

### Immunofluorescence

MDA-MB-231 and BT549 cells expressing control or CAPER shRNAs were plated at equal densities of 100,000 per well on poly-L-lysine coated coverslips in a 6-well plate (cat# 0720083, Fisher Scientific) for 48 hours. Cells were rinsed twice with 1X PBS (1 mM MgCl_2_ + 0.1 mM CaCl_2_) and fixed with 2% paraformaldehyde (PFA, cat #NC0267327, Fisher Scientific) in 1× PBS for 20 minutes at room temperature. Cells were rinsed 3 times with 1× PBS and incubated with anti-CAPER antibody (1:100) in IF buffer (1× PBS + 5% BSA + 0.5% NP40) for 45 minutes at 37° C. Secondary anti-rabbit antibody Alexa Fluor 594 (cat# A11037, Invitrogen, Carlsbad, CA) (1:500) in IF buffer was added to cells and incubated for 45 minutes at 37° C. Cells were then rinsed 3 times in 1× PBS and mounted with ProLong^®^ Gold antifade reagent with DAPI (cat# P36931, Life Technologies) and visualized using the DAPI fluorescent light cube (EVOS amep4650) and Texas Red fluorescent light cube (EVOS amep4655).

### Apoptosis assays

The Muse^®^ Cell Analyzer (EMD Millipore, Billerica, MA) and Muse^®^ Caspase-3/7 Assay kit (cat# MCH100108) were used to determine apoptotic cell death. Live and dead cells were collected and combined and cell samples were prepared and incubated with Muse^®^ Caspase-3/7 working solution in the dark for 30 minutes at 37 degrees. Next, the Muse^®^ 7-AAD working solution was added for 5 minutes. Samples were read on the Muse^®^ Cell Analyzer and results were reported as percentages of live (lower left quadrant), apoptotic (lower right quadrant), apoptotic/dead (upper right quadrant) and dead (upper left quadrant) cells.

### DNA damage assay

the Muse^®^ Cell Analyzer (EMD Millipore) and Muse^®^ H2AX Activation Dual Detection Kit (cat#MCH200101) were used to detect DNA damage. Live and dead cells were collected and combined, and cell samples were prepared and incubated with Muse^®^ H2AX antibody cocktail for 30 minutes at room temperature (in the dark). Samples were acquired on Muse^®^ Cell Analyzer and results were reported as percentages of activated H2AX (upper right quadrant).

### Cell cycle assay

The Muse^®^ Cell Analyzer (EMD Millipore) and Muse^®^ Cell Cycle Assay Kit (cat#MCH100106) were used to assess cell cycle changes following CAPER knockdown. 1 million live cells were collected and fixed with 70% ethanol for 3 hrs at −20° C and then washed with cold PBS1X. A 200 ul aliquot of fixed cells was then mixed with an equal amount of cell cycle reagent provided in the kit for 30 minutes at room temperature (in the dark). Samples were acquired on the Muse^®^ Cell Analyzer.

### Cell lines authentication

All cell lines used in this manuscript were directly purchased from ATCC and delivered to our laboratory at USciences. Cells were kept at very low passage following purchase from ATCC (<10 passages) and all experiments with CAPER ShRNA vs controls were done on passage 2, thus preventing cell phenotypes from drifting away from initial phenotype.

### Statistical analysis

Data are expressed as means ± standard error of the mean. Comparisons between more than two groups were performed using one-way ANOVA followed by a post-hoc one-sided Dunnett's multiple comparison test. Comparisons involving only two groups were conducted using two-sample *t*-tests. Statistical significance was assumed at *p <* 0.05 (^*^), *p <* 0.01(^**^) and *p <* 0.001 (^***^). All statistical analyses were performed using SAS v9.4 (SAS Institute Inc., Cary, NC).

## References

[1] Siegel RL, Miller KD, Jemal A (2018). Cancer statistics. CA Cancer J Clin.

[2] Tischkowitz M, Brunet JS, Begin LR, Huntsman DG, Cheang MC, Akslen LA, Nielsen TO, Foulkes WD (2007). Use of immunohistochemical markers can refine prognosis in triple negative breast cancer. BMC Cancer.

[3] Dent R, Trudeau M, Pritchard KI, Hanna WM, Kahn HK, Sawka CA, Lickley LA, Rawlinson E, Sun P, Narod SA (2007). Triple-negative breast cancer: clinical features and patterns of recurrence. Clin Cancer Res.

[4] Kumar P, Aggarwal R (2016). An overview of triple-negative breast cancer. Arch Gynecol Obstet.

[5] De Giorgi U, Rosti G, Frassineti L, Kopf B, Giovannini N, Zumaglini F, Marangolo M (2007). High-dose chemotherapy for triple negative breast cancer. Ann Oncol.

[6] Moran MS (2015). Radiation therapy in the locoregional treatment of triple-negative breast cancer. Lancet Oncol.

[7] Lehmann BD, Bauer JA, Schafer JM, Pendleton CS, Tang L, Johnson KC, Chen X, Balko JM, Gomez H, Arteaga CL, Mills GB, Sanders ME, Pietenpol JA (2014). PIK3CA mutations in androgen receptor-positive triple negative breast cancer confer sensitivity to the combination of PI3K and androgen receptor inhibitors. Breast Cancer Res.

[8] Ossovskaya V, Wang Y, Budoff A, Xu Q, Lituev A, Potapova O, Vansant G, Monforte J, Daraselia N (2011). Exploring molecular pathways of triple-negative breast cancer. Genes Cancer.

[9] Jung DJ, Na SY, Na DS, Lee JW (2002). Molecular cloning and characterization of CAPER, a novel coactivator of activating protein-1 and estrogen receptors. J Biol Chem.

[10] Mai S, Qu X, Li P, Ma Q, Cao C, Liu X (2016). Global regulation of alternative RNA splicing by the SR-rich protein RBM39. Biochim Biophys Acta.

[11] Mercier I, Casimiro MC, Zhou J, Wang C, Plymire C, Bryant KG, Daumer KM, Sotgia F, Bonuccelli G, Witkiewicz AK, Lin J, Tran TH, Milliman J (2009). Genetic ablation of caveolin-1 drives estrogen-hypersensitivity and the development of DCIS-like mammary lesions. Am J Pathol.

[12] Mercier I, Gonzales DM, Quann K, Pestell TG, Molchansky A, Sotgia F, Hulit J, Gandara R, Wang C, Pestell RG, Lisanti MP, Jasmin JF (2014). CAPER, a novel regulator of human breast cancer progression. Cell Cycle.

[13] Detre S, Saclani Jotti G, Dowsett M (1995). A “quickscore” method for immunohistochemical semiquantitation: validation for oestrogen receptor in breast carcinomas. J Clin Pathol.

[14] Sillars-Hardebol AH, Carvalho B, Tijssen M, Belien JA, de Wit M, Delis-van Diemen PM, Ponten F, van de Wiel MA, Fijneman RJ, Meijer GA (2012). TPX2 and AURKA promote 20q amplicon-driven colorectal adenoma to carcinoma progression. Gut.

[15] Sillars-Hardebol AH, Carvalho B, Belien JA, de Wit M, Delis-van Diemen PM, Tijssen M, van de Wiel MA, Ponten F, Meijer GA, Fijneman RJ (2012). CSE1L, DIDO1 and RBM39 in colorectal adenoma to carcinoma progression. Cell Oncol (Dordr).

[16] Song X, Zhang C, Zhao M, Chen H, Liu X, Chen J, Lonard DM, Qin L, Xu J, Wang X, Li F, O’Malley BW, Wang J (2015). Steroid Receptor Coactivator-3 (SRC-3/AIB1) as a Novel Therapeutic Target in Triple Negative Breast Cancer and Its Inhibition with a Phospho-Bufalin Prodrug. PLoS One.

[17] Roy S, Chakravarty D, Cortez V, De Mukhopadhyay K, Bandyopadhyay A, Ahn JM, Raj GV, Tekmal RR, Sun L, Vadlamudi RK (2012). Significance of PELP1 in ER-negative breast cancer metastasis. Mol Cancer Res.

[18] Shen Q, Uray IP, Li Y, Krisko TI, Strecker TE, Kim HT, Brown PH (2008). The AP-1 transcription factor regulates breast cancer cell growth via cyclins and E2F factors. Oncogene.

[19] Wang JY (2001). DNA damage and apoptosis. Cell Death Differ.

[20] Kaina B (2003). DNA damage-triggered apoptosis. critical role of DNA repair, double-strand breaks, cell proliferation and signaling. Biochem Pharmacol.

[21] Kozlov SV, Graham ME, Jakob B, Tobias F, Kijas AW, Tanuji M, Chen P, Robinson PJ, Taucher-Scholz G, Suzuki K, So S, Chen D, Lavin MF (2011). Autophosphorylation and ATM activation: additional sites add to the complexity. J Biol Chem.

[22] Short SC, Giampieri S, Worku M, Alcaide-German M, Sioftanos G, Bourne S, Lio KI, Shaked-Rabi M, Martindale C (2011). Rad51 inhibition is an effective means of targeting DNA repair in glioma models and CD133+ tumor-derived cells. Neuro Oncol.

[23] Graeser M, McCarthy A, Lord CJ, Savage K, Hills M, Salter J, Orr N, Parton M, Smith IE, Reis-Filho JS, Dowsett M, Ashworth A, Turner NC (2010). A marker of homologous recombination predicts pathologic complete response to neoadjuvant chemotherapy in primary breast cancer. Clin Cancer Res.

[24] Chen G, Yuan SS, Liu W, Xu Y, Trujillo K, Song B, Cong F, Goff SP, Wu Y, Arlinghaus R, Baltimore D, Gasser PJ, Park MS (1999). Radiation-induced assembly of Rad51 and Rad52 recombination complex requires ATM and c-Abl. J Biol Chem.

[25] Shaul Y (2000). c-Abl: activation and nuclear targets. Cell Death Differ.

[26] Meltser V, Ben-Yehoyada M, Shaul Y (2011). c-Abl tyrosine kinase in the DNA damage response: cell death and more. Cell Death Differ.

[27] Mai S, Qu X, Li P, Ma Q, Liu X, Cao C (2016). Functional interaction between nonreceptor tyrosine kinase c-Abl and SR-Rich protein RBM39. Biochem Biophys Res Commun.

[28] Welch PJ, Wang JY (1995). Abrogation of retinoblastoma protein function by c-Abl through tyrosine kinase-dependent and -independent mechanisms. Mol Cell Biol.

[29] Barila D, Rufini A, Condo I, Ventura N, Dorey K, Superti-Furga G, Testi R (2003). Caspase-dependent cleavage of c-Abl contributes to apoptosis. Mol Cell Biol.

[30] Wang JY, Naderi S, Chen TT (2001). Role of retinoblastoma tumor suppressor protein in DNA damage response. Acta Oncol.

[31] Bartkova J, Horejsi Z, Koed K, Kramer A, Tort F, Zieger K, Guldberg P, Sehested M, Nesland JM, Lukas C, Orntoft T, Lukas J, Bartek J (2005). DNA damage response as a candidate anti-cancer barrier in early human tumorigenesis. Nature.

[32] Liu Y, Burness ML, Martin-Trevino R, Guy J, Bai S, Harouaka R, Brooks MD, Shang L, Fox A, Luther TK, Davis A, Baker TL, Colacino J (2017). RAD51 Mediates Resistance of Cancer Stem Cells to PARP Inhibition in Triple-Negative Breast Cancer. Clin Cancer Res.

[33] Wang D, Du R, Liu S (2017). Rad51 inhibition sensitizes breast cancer stem cells to PARP inhibitor in triple-negative breast cancer. Chin J Cancer.

[34] Ahn SG, Kim SJ, Kim C, Jeong J (2016). Molecular Classification of Triple-Negative Breast Cancer. J Breast Cancer.

[35] Anders CK, Winer EP, Ford JM, Dent R, Silver DP, Sledge GW, Carey LA (2010). Poly(ADP-Ribose) polymerase inhibition: “targeted” therapy for triple-negative breast cancer. Clin Cancer Res.

[36] Collignon J, Lousberg L, Schroeder H, Jerusalem G (2016). Triple-negative breast cancer: treatment challenges and solutions. Breast Cancer (Dove Med Press).

[37] Locatelli MA, Aftimos P, Dees EC, LoRusso PM, Pegram MD, Awada A, Huang B, Cesari R, Jiang Y, Shaik MN, Kern KA, Curigliano G (2017). Phase I study of the gamma secretase inhibitor PF-03084014 in combination with docetaxel in patients with advanced triple-negative breast cancer. Oncotarget.

